# PCDH7 Inhibits the Formation of Homotypic Cell-in-Cell Structure

**DOI:** 10.3389/fcell.2020.00329

**Published:** 2020-05-08

**Authors:** Chenxi Wang, Ang Chen, Banzhan Ruan, Zubiao Niu, Yan Su, Hongquan Qin, You Zheng, Bo Zhang, Lihua Gao, Zhaolie Chen, Hongyan Huang, Xiaoning Wang, Qiang Sun

**Affiliations:** ^1^School of Biology and Biological Engineering, South China University of Technology, Guangzhou, China; ^2^Laboratory of Cell Engineering, Institute of Biotechnology, Beijing, China; ^3^Department of Biology, Hainan Medical University, Haikou, China; ^4^Department of Oncology, Capital Medical University, Beijing, China; ^5^Beijing Key Laboratory of Aging and Geriatrics, Chinese PLA General Hospital, Beijing, China

**Keywords:** PCDH7, cell-in-cell structure, adherens junctions, actomyosin, pMLC2

## Abstract

Though homotypic cell-in-cell (hoCIC) structures are implicated in the development and progression of multiple human tumors, the molecular mechanisms underlying their formation remain poorly understood. We found that the expression of Protocadherin-7 (PCDH7), an integral membrane protein, was negatively associated with the formation of hoCIC structures. Overexpression of *PCDH7* efficiently inhibits, while its depletion significantly enhances, hoCIC formation, which was attributed to its regulation on intercellular adhesion and contractile actomyosin as well. Via directly interacting with and inactivating PP1α, a protein phosphatase that dephosphorylates pMLC2, PCDH7 increases the level of pMLC2 leading to enhanced actomyosin at the intercellular region and compromised hoCIC formation. Remarkably, *PCDH7* enhanced anchorage-independent cell growth in a hoCIC-dependent manner. Together, we identified PCDH7 as the first *trans-*membrane protein that inhibits hoCIC formation to promote tumor growth.

## Introduction

Cell-in-cell (CIC) structures are characterized by the presence of one or more viable cells inside of another cell, which had been documented in a wide range of human tumor tissues for over a century and profoundly impact patient prognosis (Huang et al., 2015b; Fais and Overholtzer, 2018; Zhang et al., 2019). Active intercellular interactions within tumors could produce CIC structures of homotypic (between same type of cells) and/or heterotypic (between different types of cells) (Huang et al., 2015a; Ruan et al., 2019), both of which generally lead to the death of internalized cells (Fais and Overholtzer, 2018). Entosis represents for one of the best studied homotypic CIC process that kills those internalized cells non-autonomously by the outer engulfing cells, it is different from the traditional cell-autonomous death programs such as apoptosis, therefore was proposed as the type IV mechanism of cell death (Martins et al., 2017). Recent advances identified entosis as an important player in multiple biological processes, including tumor growth and evolution (Kroemer and Perfettini, 2014; Sun et al., 2014b), genome stability (Liang et al., 2020), linker cell clearance (Lee et al., 2019) and embryo implantation (Li et al., 2015) and the like.

Entosis is initiated by internalizing neighboring cells to form hoCIC structures. Adherens junction (AJ) and contractile actomyosin (CA) are two core elements controlling entotic hoCIC formation (Ning et al., 2015). While E-cadherin-mediated AJ functions to bring cells together, and establish an asymmetric cortical actomyosin by recruiting p190 RhoGAP to cell-cell contact, the contraction of actomyosin at periphery cortex drives cell internalization (Sun et al., 2014a). A group of factors, such as LPA, cholesterol, *IL-8* and *CDKN2A* that regulating hoCIC, turned out to target either AJ or CA or both (Purvanov et al., 2014; Liang et al., 2018; Ruan et al., 2018a, b). Additionally, cell internalization is also controlled at transcriptional level by MRTF and NUPR1, respectively (Cano et al., 2012; Hinojosa et al., 2017); and our recent work identified mechanical ring (MR), a ring-like structure spatially interfacing between AJ and CA, as a novel core element that couples and coordinates with AJ and CA to drive cell internalization (unpublished data). Interestingly, though E-cadherin-mediated intercellular adhesion is critical for hoCIC formation (Sun et al., 2014a; Wang et al., 2015), an integral membrane protein that negatively regulates cell-cell adhesion and hoCIC formation remains to be identified.

To explore the molecular control of hoCIC formation, we previously performed expression profiling analysis of a panel of cells differing in their abilities to form hoCIC structures (Ruan et al., 2018a). Protocadherin-7 (PCDH7), an integral membrane protein belonging to cadherin superfamily, was found to negatively regulate the formation of hoCIC structures that contribute to anchorage-independent cell growth. This effect is correlated with its ability to attenuate cell-cell adhesion and increase junctional pMLC2 via interacting with protein phosphatase 1α (PP1α). Thus, this work reports the first *trans-*membrane protein that suppresses hoCIC formation to promote tumor growth.

## Materials and Methods

### Cells and Culture Conditions

Six breast cancer cell lines (MCF7, FENT, FK12, ZR75-1, MDA-MB-436, and MDA-MB-436-2) and HEK293FT cells used in this study were from our lab. FK12 and FENT were two monoclonal cell lines isolated from ZR75-1. MDA-MB-436-2 is a cell clone isolated from MDA-MB-436. All of them were cultured in DMEM (MACGENE Technology Ltd., Beijing, China) supplemented with 10% fetal bovine serum (PAN-Biotech, Germany), 100 units/ml of penicillin and streptomycin (MACGENE Technology Ltd., Beijing, China). All cells were maintained in the humidified incubator of 5% CO2 at 37°C.

### Antibodies and Chemical Reagents

The primary antibodies with working dilution factors, company source and catalog number include: anti-PCDH7 (1:500 for western blot (WB) and 1:200 for immunofluorescence (IF); Abcam; ab139274), anti-E-cadherin (1:1000 for WB; BD; 610181), anti-E-cadherin (1:200 for IF; CST; #3195S),anti-α-catenin (1:500 for WB; BD; 610193), anti-β-catenin (1:2000 for WB; BD; 610154), anti-γ-catenin (1:4000 for WB; BD; 610253), anti-p120-catenin (1:1000 for WB; BD; 610133), anti-pMLC2 (1:1000 for WB and 1:200 for IF; CST; #3671S), anti-PP1α(1:1000 for WB, 1:200 for IF and 1:100 for IP; Santa Cruz; sc-7482), anti-MLC2 (1:1000 for WB; CST; #3672s), anti-α-tubulin (1:1000 for WB; Proteintech; 11224-1-AP), anti-Flag-Tag (1:125 for Co-immunoprecipitation; Abbkine; BB-A02010), anti-Flag-Tag (1:500 for WB; Proteintech; 20543-1-AP), anti-mCherry (1:4000 for WB; GeneTex; GTX128508), anti-IgG (1:100 for IP; Abcam; ab102455). Secondary antibodies include Alexa Fluor 568 anti-mouse (1:500; Invitrogen; A11031), Alexa Fluor 568 anti-rabbit (1:500; Invitrogen; A11036), Alexa Fluor 647 anti-mouse (1:500; Invitrogen; A21236) and Alexa Fluor 647 anti-rabbit (1:500; Invitrogen; A21245). anti-rabbit IgG HRP (1:3000; CST; #7074), anti-mouse IgG HRP (1:3000; CST; #7076). Hoechst 33342 (H3570, Thermo) was used for cell nuclear staining. Y27632 (ROCK inhibitor, TOCRIS) was used at working concentration of 10 μM.

### Constructs and Stable Cell Lines

cDNA was generated from MDA-MB-436 using the TransScript One-Step gDNA Removal and cDNA Synthesis SuperMix (TransGen Biotech). *PCDH7* isoforms A-D were amplified from cDNA and subsequently cloned into the cloning vector pGEM-T (Promega) and confirmed by sequencing. *PCDH7* isoforms were then subcloned into retroviral vector pQCXIP-EGFP-N1 at *Eco*RI/*Age*I sites. *PCDH7A, PCDH7C*, and *PCDH7D* were subcloned into pcDNA3.1-3 × Flag to generate pcDNA3.1-PCDH7-A-3 × Flag, pcDNA3.1-PCDH7-C-3 × Flag and pcDNA3.1-PCDH7-D-3 × Flag. The hairpin target sequences of *PCDH7* were: shRNA1 (5′-CCAAGCTATGAAATTAGCAAA-3′), shRNA2 (5′-CGTGCTTGACATCAACGACAA-3′). They were subcloned into lentiviral vector pLVX at *Eco*RI/*Bam*HI sites. Plasmid pmCherry-PP1α-N1 was purchased from Youbio biological technology. Stable cell lines were established by virus infection as described (Wang et al., 2015). Virus-infected tumor cells were selected with puromycin (1 μg/mL) for 7–14 days.

### siRNA Transfection

About 2.5 × 10^5^ cells were plated per well in 6-well plate cells and cultured for 16 h at 37°C before transfected with siRNAs for *PCDH7* (1#: sense-5′-CCAAGCUAUGAAAUUAG CAAATT-3′, antisense-5′-UUUGCUAAUUUCAUAGCUUGGTT-3′; 2#: sense-5′-GCUGGCAUUAUGACGGUGAUUTT-3′, anti- sense-5′-AAUCACCGUCAUAAUGCCAGCTT-3′) and *PP1*α (1#: sense-5′-CCGCAUCUAUGGUUUCUACTT-3′, antisense- 5′-GUAGAAACCAUAGAUGCGGTT-3′; 2#: sense-5′-CAUCU AUGGUUUCUACGAUTT-3′, antisense-5′-AUCGUAGAAAC CAUAGAUGTT-3′) and negative control siRNA (synthesized by GenePharma, China) using Lipofectamine RNAiMAX (Invitrogen, California, United States) according to the manufacturer’s recommendations. The knockdown efficiency was measured by qPCR 36 h after transfection.

### Quantitative Real-Time PCR

Quantitative real-time PCR (qPCR) was performed as described (Ruan et al., 2018a). qPCR was performed at 95°C for 30 s, then 40 cycles of 95°C for 5 s, 60°C for 10 s, and then 72°C for 15 s. Fold changes were calculated using the 2^–ΔΔCt^ method and were expressed as means ± SD of triplicate quantification. The following primer pairs were used:

*HPRT*: forward 5′-AGGCCATCACATTGTAGCCCTCTGT-3′
reverse 5′-TACTGCCTGACCAAGGAAAGCAAAGT-3′;
*PCDH7*: forward 5′-CAATGCTCCCACAGTTACCCT-3′
reverse 5′-ACTGTCATTCACTTGCACCAC-3′;
*PP1*α: forward: 5′-CCTCCAGAGAGCAACTACCTCTTC-3′
reverse 5-ACGTCTTCCACAGTTTGATGTGTTGT-3′.

### Cell-in-Cell Formation Assay

Cell-in-cell formation assay was performed as described (Wang et al., 2015). Briefly, about 2.5 × 10^5^ cells were suspended in 6-well plate precoated with 0.5% soft agar for 6 h. Cells were then collected and centrifuged at 1000 rpm for 3 min. After that cytospins were made by centrifuging at 400 rpm for 3 min (WESCOR, United States). Then, cells were fixed and immunostained with DAPI to quantify cell-in-cell structures. Structures with more than half of invading cell internalized were considered as CIC structures.

### Cell Competition Assay

For identity analysis of winners and losers, equal number of cells from different cell lines, stained with green or red CellTrackers, were mixed together and suspended in 6-well plate precoated with 0.5% soft agar for 4–5 h at 37°C. Cells were then collected and centrifuged at 1000 rpm for 3 min. Cytospins were fixed and mounted as above in CIC formation assay. To assess the effects of gene expression on cell identity, CIC structures formed between green cells and red cells were analyzed.

### Immunofluorescence and Immunoblotting

For immunostaining, cells were first fixed with 4% PFA or 10% TCA for 10 min at room temperature, then permeablized in 0.15% Triton X-100 for 3 min followed by blocking with 5% BSA for 1 h. The Primary antibodies were incubated with samples in a humidifed box at 4°Covernight followed by three 10-min washes with PBS the next day. Secondary antibodies were used for 1 h at room temperature followed by three 10 min washes with PBS. Images were captured by Ultraview Vox confocal system (Perkin Elmer) on Nikon Ti-E microscope. Immunoblotting was performed as previously described (Liang et al., 2018). Briefly, Cell lysis was performed in ice-cold RIPA buffer with protease inhibitor cocktail (1:100; CWBiotech, Beijing) and phosphatase inhibitor cocktail (1:100; CWBiotech, Beijing), and quantified using the BCA Protein Assay (Thermo Scientific) followed by SDS-PAGE and then transferred onto 0.2 μm polyvinylidene fluoride membrane (Merck Millipore Ltd.), after which antibodies were used to probe specified protein.

### Clustering Assay

In order to detect the ability of cells forming cluster, 2.5 × 10^5^ cells were suspended in 6-well plates pre-plated with 0.5% soft gar for 6 h at 37°C. Cytospins were made and stained as above in CIC formation assay to quantify cell cluster. Cell colony that contains more than 6 cells was considered to be one cell cluster. Cells in cluster rate (%) = (number of tumor cells involved in cell cluster/number of total tumor cells counted) × 100%.

### Compression Assay

Compression assay was performed as described (Miao et al., 2017). Briefly, the transparent agarose gel (2% weight) was prepared as described before experiment. Then, about 2.0 × 10^5^ cells were suspended in 12-well glass plate and images of XYZ plane were quickly acquired by using Ultra VIEW^®^ VOX Confocal Microscope (PerkinElmer, United States). After that, most of the cells condensed to the bottom of the chamber and weakly adhered onto the glass substrate. A piece of agarose gel slightly smaller than the well of glass plate was carefully loaded into the chamber without folding using a tweezer. Images were quickly acquired again as before. The average pressure of a single cell is W^∗^L^∗^R^∗^R^∗^M^∗^g/(S^∗^N). W is the width of image in microscope; L is the length of image; R is the microscopy coefficient; M is the weight of agarose gel; *g* = 9.8 N/kg; S is the size of agarose gel; M^∗^g/S is the gravity density; N is the number of cells in image. The height change rate is (Z_0_-Z_N_)/Z_0_^∗^100%. Z_0_ is the average value of original cells height (*n* > 30), Z_N_ is the average value of final cells height.

### Co-immunoprecipitation Assay

For exogenous protein immunoprecipitation (IP) assay, about 1 × 10^6^ HEK293FT cells were plated per well in 6-well plates and cultured for 16 h at 37°C before transfected with different plasmids. Two days later, cells were collected for subsequent experiments. MDA-MB-436-2 cells were used to perform endogenous protein IP assay. HEK293FT cells and MDA-MB-436-2 cells were lysed by the ice cold IP lysis buffer (20 mM Tris, 0.1 M NaCl, 0.1% NP40, 5 mM EDTA in ddH2O and PH = 8) with phosphatase inhibitor cocktail (CWBiotech, Beijing) and protease inhibitor cocktail (CWBiotech, Beijing), and IP experiment was performed using the protein A/G agarose (Beyotime Biotechnology). Then, lysates were further cracked with ultrasound (power 40%, work 6 s, stop 9 s, 5 times in total). After being centrifuged at 12,000 rpm for 10 min, the supernatant was collected, and a small amount of which was for input. The remaining supernatant was blocked with 20 μl protein A/G beads (pre-washed with cold IP lysis buffer) for 1 h. Flag-Tag (Abbkine) or anti-PP1α or anti-IgG was incubated with protein lysate removed protein A/G agarose at 4°C overnight. The next day, add 30 μl protein A/G beads into the protein lysate and continue to incubate for 2 h, and beads were washed extensively with cold IP lysis buffer. IP products were harvested using denaturing elution and subjected to western blot analysis to detect protein-protein interactions.

### Growth Assay

Cells were seeded at 5000 cells/well (MCF7 and derivatives) or 3000 cells/well (MDA-MB-436 and derivatives) in 96 well plates. After incubation for indicated periods, the MTT kit (GEN-VIEW) was used according to the manufacturer’s recommendation. Each assay was conducted in triplicate.

### Anchorage-Independent Growth

Anchorage-independent growth was performed as described (Sun et al., 2014a). Briefly, about 5000 cells were embedded into 0.4% (media: 2.0% agarose = 4:1) low melting agarose (Solarbio), and plated onto 0.5% agarose pads in 6-well plate. After the agarose solidified in room temperature, 1 ml media with or without Y27632 was added. Medium was changed every 3 days for 3 weeks. Finally, Colonies were stained with 0.02% iodonitrotetrazolium chloride (Sigma) and quantified using ImageJ software (NIH).

### Statistical Analysis

Data were displayed as mean ± standard deviation (SD). Student’s *t*-test was performed to examine statistically significant differences when comparing two groups, and Dunnett-t test was used when comparing more than two groups. Statistical differences were performed with SPSS 22.0 software. *P* < 0.05 was considered statistically significant.

## Results

### PCDH7 Is a Negative Regulator of Homotypic Cell-in-Cell Formation

**FIGURE 1:**
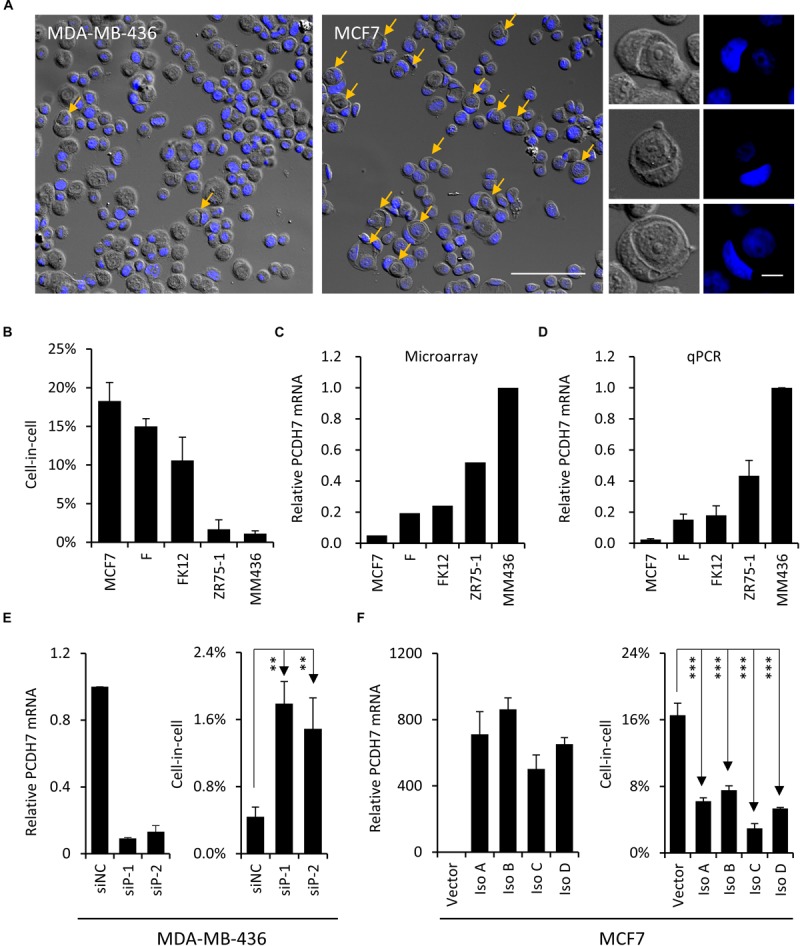
PCDH7 inhibits homotypic cell-in-cell formation. **(A)** Representative cytospin images for MCF7 and MDA-MB-436 cells. Representative images for typical CIC structures of different stages are shown in right. Yellow arrows indicate hoCIC. The nuclei were stained with DAPI (blue). Scale bar: 100 μm (left) and 10 μm (right). **(B)** The frequencies of hoCICs formation in different breast tumor cell lines. Data are mean ± SD of triplicate experiments. **(C,D)** *PCDH7* expression in microarray **(C)** and determined by quantitative RT-PCR **(D)**. Data are mean ± SD of triplicate experiments. **(E)** *PCDH7* knock-down (left) results in increased CIC formation (right) in MDA-MB-436 cells. Data are mean ± SD of triplicate experiments. ^∗∗^*P* < 0.01. **(F)** Overexpression of *PCDH7* isoforms (left) inhibits CIC formation (right) in MCF7 cells. Data are mean ± SD of triplicate experiments. ^∗∗∗^*P* < 0.001.

To explore the molecular mechanisms underlying the formation of hoCIC structures, we selected a panel of breast cancer cell lines for expression profiling (Ruan et al., 2018a). These 5 lines of cells are different in their abilities to form hoCIC structures (Figures 1A,B), with MCF7 displaying highest hoCIC frequency (Figures 1A middle, 1B). Among the candidate genes identified from expression profiling, *PCDH7*’s expression level (Figure 1C), validated by quantitative real-time PCR (qPCR) (Figure 1D), is negatively correlated with hoCIC frequency (Figures 1B–D), suggesting that *PCDH7* might be a negative regulator of hoCIC formation.

To test the idea, RNA interference experiment was performed to knock down *PCDH7* expression in MDA-MB-436 cells, where *PCDH7* is expressed at the highest level among all lines of cells examined (Figures 1C,D). As a result, *PCDH7* depletion increased hoCIC formation significantly (Figure 1E). To further confirm the effects of *PCDH7* on hoCIC formation, we overexpressed all four isoforms of *PCDH7* in MCF7 cells, where *PCDH7* is expressed at the lowest level (Figures 1C,D). Expression of all of the isoforms effectively inhibits hoCIC formation in MCF7 cells (Figure 1F). Collectively, these data support that *PCDH7 i*s a negative regulator of hoCIC formation.

### PCDH7 Inhibits Intercellular Adhesion

**FIGURE 2:**
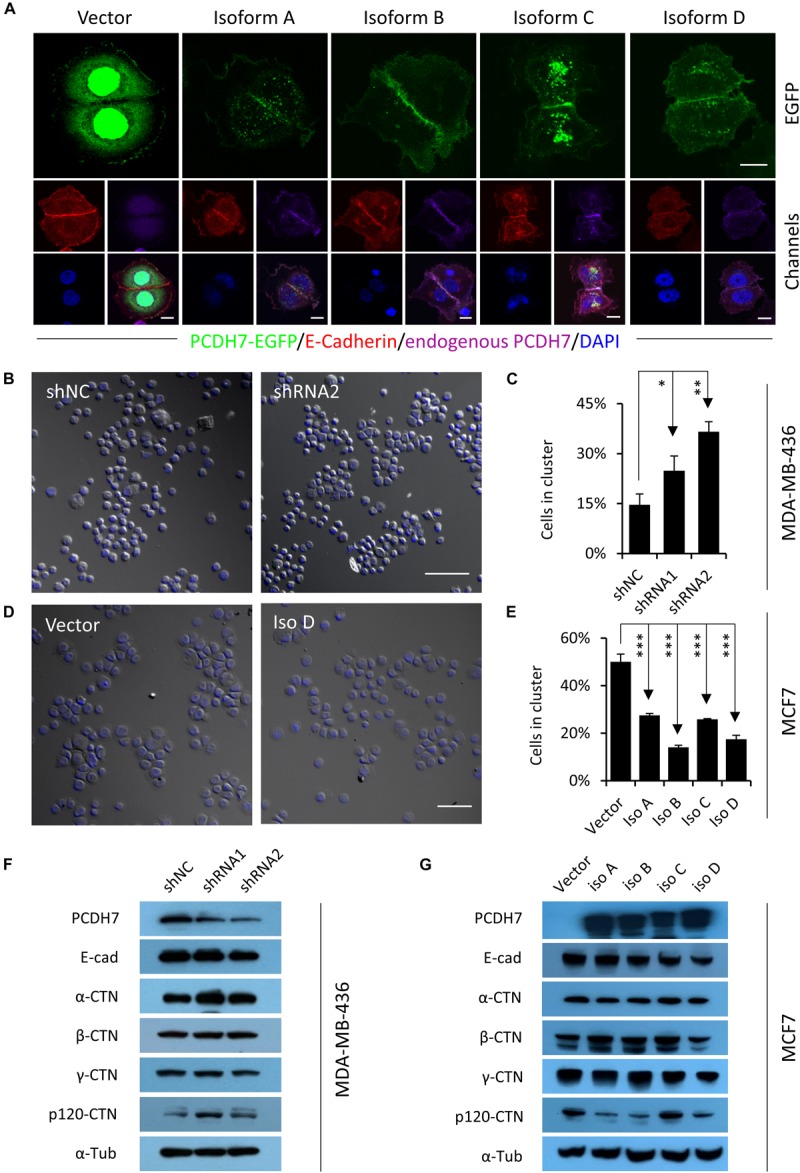
PCDH7 inhibits intercellular adhesion. **(A)** Subcellular localization of PCDH7 isoforms in MCF7 cells. Cells expressing EGFP-tagged PCDH7 isoforms (green) were stained with anti-E-cadherin (red) and anti-PCDH7 (purple) antibodies. Scale bars: 10 μm. **(B)** Representative images for cluster formation of *PCDH7*-depleted MDA-MB-436 cells. The nuclei were stained with DAPI. Scale bar: 100 μm. **(C)** Quantification of cells forming clusters in *PCDH7*-depleted MDA-MB-436 cells. Data are mean ± SD of triplicate experiments. ^∗^*P* < 0.05; ^∗∗^*P* < 0.01. **(D)** Representative images for cluster formation of MCF7 cells overexpressing PCDH7D. The nuclei were stained with DAPI. Scale bar: 100 μm. **(E)** Quantification of cells forming clusters in MCF7 cells overexpressing *PCDH7*. Data are mean ± SD of triplicate experiments. ^∗∗∗^*P* < 0.001. **(F,G)** Expression of adhesion molecules upon *PCDH7* depletion **(F)** or overexpression **(G)** as detected by Western blot.

To explore the potential mechanisms whereby *PCDH7* affecting hoCIC formation, we expressed EGFP-tagged *PCDH7* in MCF7 cells, and found that all four exogenous isoforms co-localized with endogenous PCDH7 and E-cadherin, the critical mediator of hoCIC formation, at cell-cell contacts (Figure 2A). This subcellular localization suggests that PCDH7 may affect intercellular adhesion. We therefore examined this idea by cluster assay. As shown in Figures 2B,C, cells with *PCDH7* depletion tend to cluster together as compared with control cells. Meanwhile, cells overexpressing *PCDH7* formed significantly less and smaller clusters than did control cells (Figures 2D,E). These data support that PCDH7 functions to inhibit intercellular adhesion and consequent hoCIC formation, in agreement with which, PCDH7 expression tends to negatively regulate the level of p120 catenin, an essential component of adherens junction prerequisite for hoCIC formation (Figures 2F,G; Sun et al., 2014a).

### PCDH7 Enhances Actomyosin Contraction and Cell Stiffness

**FIGURE 3:**
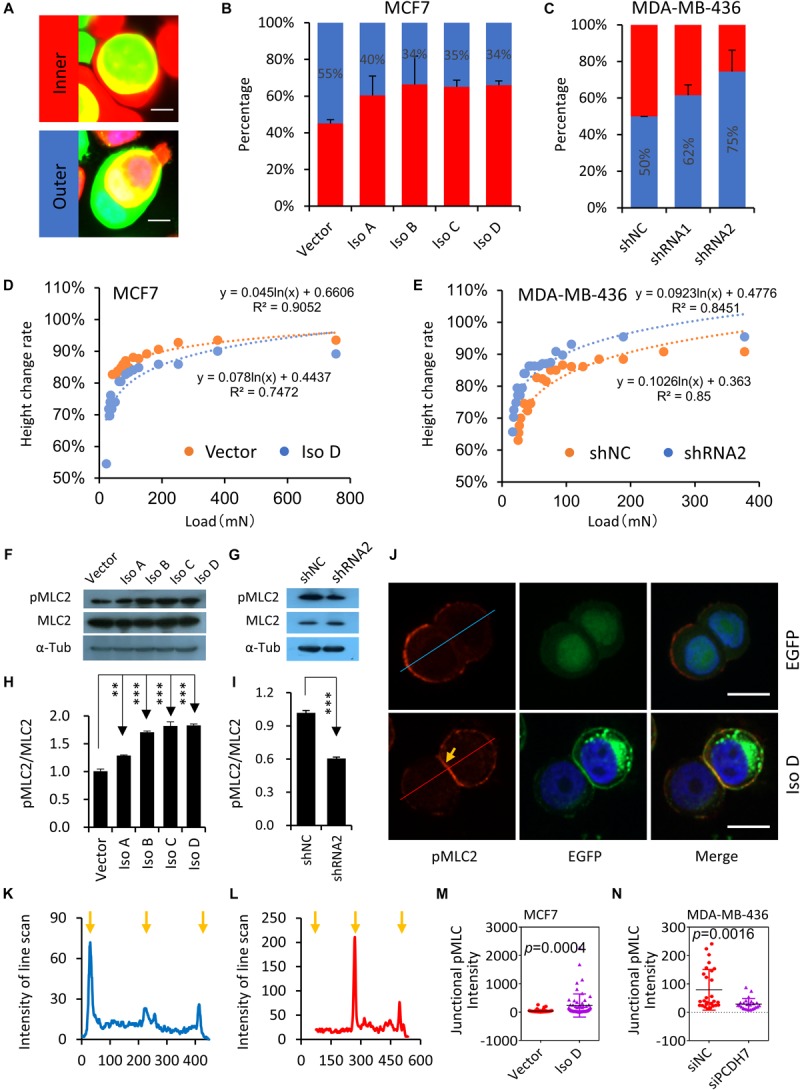
PCDH7 enhances actomyosin contraction and cell stiffness. **(A)** Images depicting the relative identities of EGFP-expressing cells. Scale bar: 10 μm **(B)** *PCDH7* overexpression (EGFP +) promotes cells internalization as inner cell. *n* > 60 CIC structures each. Data are mean ± SD of triplicate experiments. *P* < 0.05 for isoforms compared with control. **(C)** *PCDH7* depletion (EGFP +) confers cells the identity of outer cell. *n* > 60 CIC structures each. Data are mean ± SD of triplicate experiments. *P* < 0.05 for two hairpins compared with control. **(D,E)** Deformation of cells with *PCDH7* overexpression **(D)** or depletion **(E)** as determined by compression assay. **(F,G)** Expression of pMLC2 and MLC2 upon *PCDH7* overexpression **(F)** or depletion **(G)**. **(H,I)** Quantification of the ratio of pMLC2/MLC2 in **(F)** or **(G)**, respectively. ^∗∗^*P* < 0.01; ^∗∗∗^*P* < 0.001. **(J)** Representative images for pMLC2 staining (red) in MCF7 cells. Arrow indicates junctional pMLC2. Scale bar: 15 μm. **(K,L)** Relative intensity of pMLC2 by line scan analysis. **(K)** and **(L)** are corresponding to upper (EGFP) and lower (Iso D) panels, respectively. Arrows indicate cell boundaries. **(M,N)** Quantification of junctional pMLC2 intensity upon *PCDH7* overexpression **(M)** or depletion **(N)**. *n* = 24∼60 junctional sites.

By checking the CIC structures, we found that cells overexpressing *PCDH7* were preferentially internalized as inner cells, which is true for all PCDH7 isoforms (Figures 3A,B), indicating that *PCDH7 e*xpression may confer inner/loser identity to cells. Consistently, *PCDH7* depletion prevents cells from internalization as loser (Figure 3C). Since we previously showed that cells’ identities during CIC formation were dictated by relative stiffness of paired cells (Sun et al., 2014b), we therefore measured the stiffness of *PCDH7*-expressing cells by agarose compression assay (Miao et al., 2017). In agreement with the identity analysis, *PCDH7* depleted cells are easier, while *PCDH7* expressing cells are harder, to be deformed upon agarose loading (Figures 3D,E). This is associated with a paralleled change in the level of pMLC2, a molecular readout of actomyosin contraction (Figures 3F–I), suggesting that PCDH7 may regulate pMLC2 and consequent actomyosin contraction, the key controller of cell stiffness and internalization (Sun et al., 2014b). Besides, we found that PCDH7 seems to spatially increase pMLC2 at cell-cell contact (Figures 3J–N), which is known to prevent hoCIC formation (Sun et al., 2014a; Ning et al., 2015). Together, our data suggest that PCDH7 may inhibit hoCIC formation by upregulating pMLC2, specifically at cell-cell contact where PCDH7 is highly enriched.

### PCDH7-PP1α Axis Inhibits Homotypic Cell-in-Cell Formation

**FIGURE 4:**
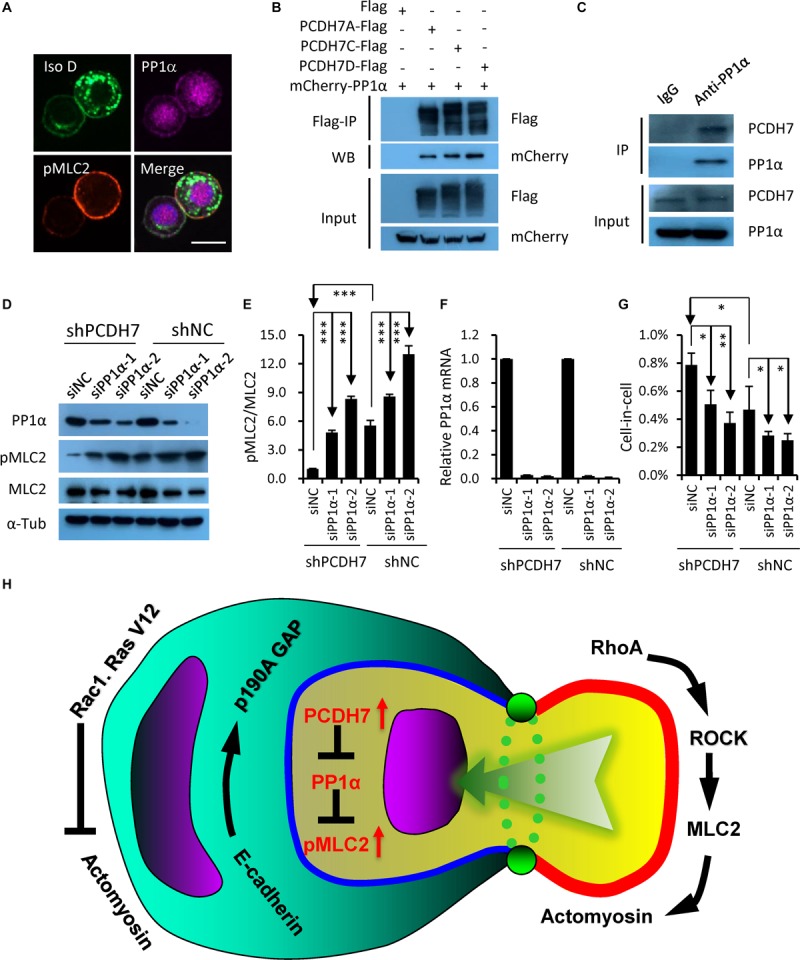
PCDH7 interacts with PP1α to inhibit homotypic cell-in-cell formation. **(A)** PCDH7 co-localizes with PP1α at cell-cell contact. Scale bars: 15 μm. **(B,C)** PP1α interacts with PCDH7 as determined by co-immunoprecipitation assay in HEK293FT cells for overexpressed proteins **(B)** or MDA-MB-436-2 cells for endogenous proteins **(C)**. **(D)** Increased pMLC2 expression upon PP1α knockdown as determined by Western blot in MDA-MB-436 cells. **(E)** Quantification of pMLC2/MLC2 in **(D)**. ^∗∗∗^*P* < 0.001. **(F)** Relative mRNA level of PP1α upon knockdown in MDA-MB-436 cells. Data are mean ± SD of triplicate experiments. **(G)** PP1α knockdown inhibits CIC formation in MDA-MB-436 cells. Data are mean ± SD of triplicate experiments. ^∗^*P* < 0.05; ^∗∗^*P* < 0.01. **(H)** Updated working model for entotic CIC formation regulated by *PCDH7* and *PP1*α.

Since PCDH7 was identified to be able to inhibit PP1α, a protein phosphatase that could target pMLC2 for de-phosphorylation (Yoshida et al., 1999; Ma et al., 2015), we hypothesized that PP1α may be involved in the *PCDH7*-regulated hoCIC formation. Consistent with the idea, PP1α was found to be colocalized with PCDH7 at cell-cell contact, where pMLC2 is enriched (Figure 4A), and immunoprecipitation confirmed the interaction of PCDH7 with PP1α (Figures 4B,C). Moreover, PP1α knockdown by RNA interference resulted in increased pMLC2 and compromised CIC formation (Figures 4D–G). Thus, our results, together with published data (Yoshida et al., 1999; Ma et al., 2015), support a model that PCDH7 inhibits PP1α to upregulate pMLC2 and actomyosin contraction at cell-cell contact, which prevents the establishment of asymmetric/polarized pMLC2 pattern from cell periphery toward cell-cell contact required for hoCIC formation, leading to the inhibited formation of CIC structures (Figure 4H).

### Cell-in-Cell Formation Is Involved in Anchorage-Independent Cell Growth Regulated by PCDH7

**FIGURE 5:**
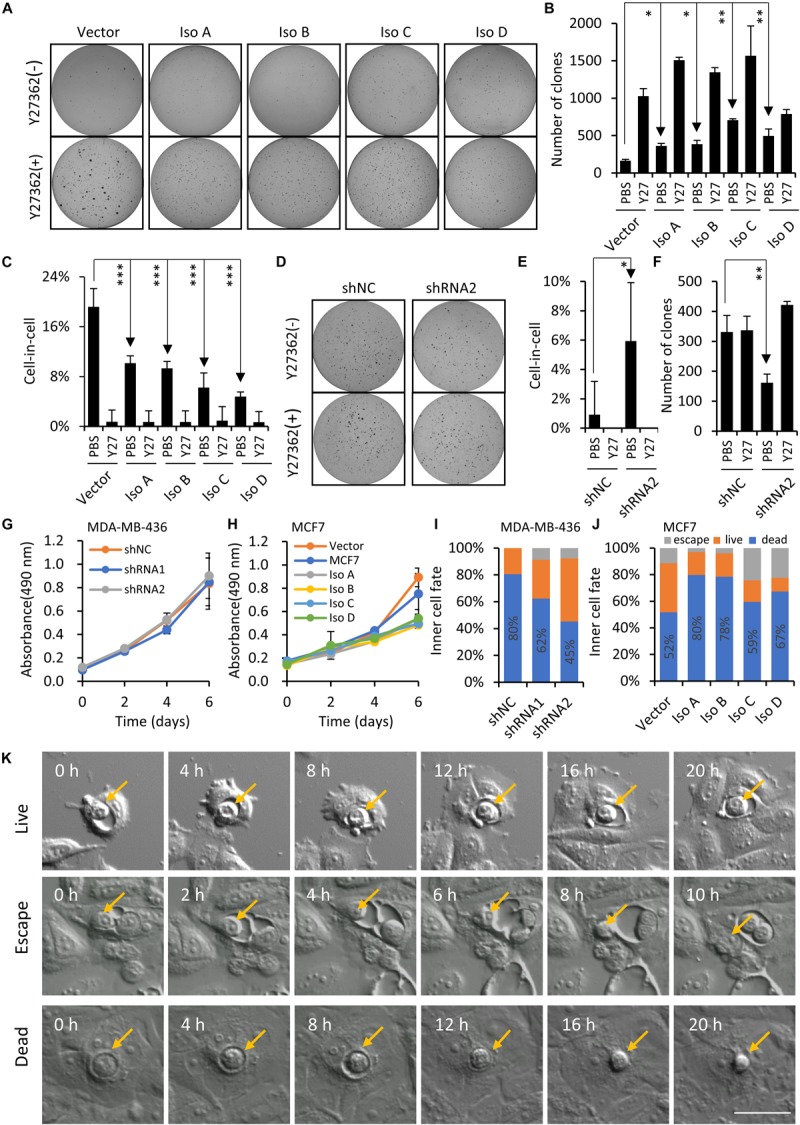
PCDH7 enhances anchorage-independent cell growth. **(A–C)** *PCDH7* overexpression promotes anchorage-independent colony formation of MCF7 cells in soft agar, which is associated with inhibited CIC formation. Y27: Y27632. Data are mean ± SD of triplicate experiments. ^∗^*P* < 0.05; ^∗∗^*P* < 0.01; ^∗∗∗^*P* < 0.001. **(D–F)** *PCDH7* depletion suppresses colony formation of MDA-MB-436 cells in soft agar in a CIC-dependent manner. Y27: Y27632. Data are mean ± SD of triplicate experiments. ^∗^*P* < 0.05; ^∗∗^*P* < 0.01. **(G,H)** Growth curves for *PCDH7*-depleted **(G)** or -overexpressing **(H)** cells cultured under adherent condition. Data are mean ± SD of triplicate experiments. **(I,J)** Inner cell fate analysis in cells with *PCDH7* depletion **(I)** or overexpression **(J)**. *n* > 40 each. **(K)** Representative image sequences showing three major fates of internalized cells. Scale bar: 50 μm.

To extend our finding on PCDH7 and hoCIC formation, we next examine the regulation of hoCIC formation by PCDH7 in the context of anchorage-independent growth in soft agar, an assay for transformed cell growth, under which condition hoCIC formation could be induced. As shown in Figure 5A, overexpression of PCDH7 isoforms significantly increases colony formation, which is associated with inhibited hoCIC formation (Figures 5B,C). This phenotype is confirmed in PCDH7-depleted cells, where increased CIC formation was observed together with decreased colony formation (Figures 5D–F). The influences of PCDH7 on cell proliferation is unlikely to be responsible for altered colony formation in soft agar (Figures 5G,H). Instead, hoCIC formation may play important role in the colony growth regulated by PCDH7 as blocking CIC formation by Y27632, the ROCKs inhibitor, completely rescued colony formation suppressed by PCDH7 depletion (Figures 5D–F). Also, the contribution of CIC structures to anchorage-independent cell growth regulated by PCDH7 may not be explained by changed inner cell fate, as PCDH7 tends to promote inner cell death (Figures 5I–K) which is conceivably suppressive, but not promotive, for cell growth.

## Discussion

The CIC structures had been extensively documented in almost every types of human cancers examined (Fais and Overholtzer, 2018). Although it was known for long time that multiple subtypes of CIC structures may arise from complex interactions between cells within the heterogeneous tumor mass, it was until recently that the CIC subtypes were finely profiled by a method called “EML” multiplex staining. In this method, the epithelial cells, macrophages and leukocytes were labeled with the corresponding antibodies, respectively, so that the cell type involving in CIC formation could be easily identified by the fluorophores tagged to the antibodies (Huang et al., 2015a). Currently, the application of the EML method had identified 5 different types of CIC structures up to date, including TiT (tumor cell inside tumor cell), MiT (macrophage in tumor cell), LiT (leukocyte in tumor cell), TiM (tumor cell in macrophage) and LiM (leukocyte in macrophage). Out of the 5 subtypes identified, TiT is hoCIC structure while the rest 4 subtype are heterotypic CIC structure (heCIC) (Huang et al., 2015a; Zhang et al., 2019). Subtyped CIC structures, particularly the hoCIC subtype, were found to be associated with higher tumor stage and/or patient survival in several types of cancers including head and neck squamous carcinoma, rectal cancer (Schwegler et al., 2015), mesothelioma (Matsumoto et al., 2013), and breast cancer (Liang et al., 2018; Zhang et al., 2019). Despite of the broad implications of hoCIC structures in human cancers, the mechanisms underlying their formation remain to be explored.

Our previous work indicates that the formation of hoCIC structures strictly requires AJ mediated by E-cadherin (Sun et al., 2014a), a member of classical cadherins. In this work, we found that PCDH7, a member of protocadherin family (Angst et al., 2001), negatively regulated the same process. It’s interesting that one biological process is regulated differently by two types of cadherins within one superfamily. This may reflect a linage-specific evolution of cadherin molecules for the related functions. Physiologically, E-cadherin is expressed strictly in epithelial cells while most protocadherin including PCDH7 are primarily expressed in non-epithelial tissues such as nervous system though they both regulate intercellular adhesion. However, they function differently in a specified context such as cancer cells, where PCDH7 attenuates cell-cell adhesion mediated by E-cadherin (Figures 2B–E). This may be attributed to their structural differences. Although both E-cadherin and PCDH7 are type I transmembrane proteins that contain multiple extracellular cadherin (EC) repeats, PCDH7 doesn’t have intracellular catenin-binding domain that signals to actin cytoskeleton, therefore integrates into distinct signaling pathways to regulated cell adhesion. As for the process of hoCIC formation, PCDH7 functions to inhibit PP1α, a well-established phosphatase of pMLC2, to increase actomyosin contraction at cell-cell contact that prevents active cell invasion. It bypasses the way of actomyosin regulation by E-cadherin who recruits p190 RhoGAP to inactivate upstream RhoA at cell-cell contact to promote cell internalization. Thus, PCDH7 could inhibit hoCIC formation in the presence of E-cadherin. Intriguingly, while previous study showed that PP1α could bind directly to the RVTF motif of the cytoplasmic region of PCDH7c (Yoshida et al., 1999), our data indicated that not only PCDH7c and PCDH7d that contain RVTF motif, but also PCDH7a that doesn’t have a RVTF motif, could co-immunoprecipitation with PP1α (Figures 4B,C). Given that PCDH7a inhibits CIC formation in an efficacy comparable to PCDH7c and PCDH7d, we consider that *PCDH7* isoforms may indirectly interact with PP1α, which is independent of RVTF motif, to form a complex that is involved in the regulation of CIC formation.

The cluster assay was generally employed to evaluate the strength of intercellular adhesion that is controlled by multiple adhesive structures. E-cadherin mediated AJ is probably one of the most representative adhesive structures that controls epithelial cell adhesion (Leckband and Rooij, 2014). Our previous work, and others’ as well (Overholtzer et al., 2007), demonstrated that the structurally and functionally intact AJ is prerequisite for the formation of hoCIC structures, the depletion of any AJ components such as E-cadherin or α-catenin may completely compromise intercellular adhesion and hoCIC formation, and restoring their expression could efficiently induce hoCIC formation in the corresponding molecules-null cells (Sun et al., 2014a; Wang et al., 2015). It’s conceivable that cell clustering would be tightly correlated with cells’ ability to form hoCIC structures. Consistent with this idea, molecules increasing cell clustering also promote hoCIC formation. For example, IL-8, a cytokine that enhances cell clustering, significantly increased hoCIC formation in MDA-MB-436 cells (Ruan et al., 2018a). Therefore, cell clustering assay might be a simple way to read out hoCIC formation in epithelial cells. It should be noted that the rule may not be applicable to the non-epithelial cells, as their intercellular adhesion is mediated by other adhesive molecules such as N-cadherin in fibroblast. In this case, hoCIC is rarely formed despite that the fibroblast cells can efficiently form big cluster.

*PCDH7* was shown to promote transformation and metastasis in several types of cancers including lung, prostate and breast cancer (Li et al., 2013; Chen et al., 2016; Zhou et al., 2017; Shishodia et al., 2019). Nevertheless, the underlying cellular mechanisms are unclear. It’s assumed that *PCDH7* may promote cell proliferation by enhancing RTK-ERK signaling via interacting with SET complex, however, with direct evidences not shown (Zhou et al., 2017; Shishodia et al., 2019). We demonstrate here that *PCDH7* could significantly increase transformed tumor cell growth in anchorage-independent condition. This works in a CIC-dependent manner, and has little association with changed cell proliferation (Figure 5). Therefore, PCDH7 may promote tumor growth via promoting cell proliferation, and inhibiting hoCIC formation as well. It’s interesting that PCDH7 expression also increase the death of internalized cells (Figures 5I–K), which is in line with its function to confer cells loser identity by enhancing actomyosin contraction (Figure 3). To our best knowledge, *PCDH7* is the first molecule that regulates hoCIC formation and subsequent death in two functionally opposite ways. The underlying biological significance and molecular mechanisms warrant further investigation.

It should be noted that PCDH7 seems to be a typical protein that functions in a context-dependent manner. For example, PCDH7 was found to promote cell proliferation in MDA-MB-231 breast cancer cells (Li et al., 2013), but failed to do so in BGC-823 and MKN-45 gastric cancer cells (Chen et al., 2017), and MCF7 and MDA-MB-436 breast cancer cells in this study (Figures 5G,H). Similarly, while we demonstrate here that PCDH7 has little impacts on the expression level of E-cadherin in MCF7 and MDA-MB-436 cells (Figures 2F,G), Chen et al. (2017) reported that E-cadherin expression was significantly downregulated upon *PCDH7* depletion in gastric cancer cells. Overexpression of PCDH7a and PCDH7b was shown to be able to promote cell-cell adhesion in fibroblast cells (Yoshida, 2003), but inhibit cell clustering in breast cancer cells in this study. Moreover, while PCDH7 expression was found to be upregulated in lung and breast cancer and associated with poor clinical outcome (Chen et al., 2016; Zhou et al., 2017), decreased PCDH7 expression was documented in bladder and gastric cancers that unfavorably impact patient survival (Beukers et al., 2013; Chen et al., 2017). Together, these data suggest that the exact roles and underlying mechanisms of *PCDH7* in hoCIC formation and human cancers may only be addressed in a confined model system.

In summary, we have identified PCDH7 as a novel, and probably the first to our best knowledge, transmembrane protein that negatively regulated the formation of hoCIC structures by entosis. And PCDH7 prevents cells from internalization into their neighbors by locally inactivating PP1α, a protein phosphatase that could target pMLC2 for de-phosphorylation, to increase actomyosin contraction at cell-cell contact.

## Data Availability Statement

All datasets generated for this study are included in the article/supplementary material.

## Author Contributions

QS conceived the project. CW, AC, and BR designed the experiments and analyzed data. CW performed the experiments. ZN, YS, and HQ provided assistance in imaging analysis. YZ and BZ provided assistance in data analysis. XW, LG, ZC, and HH provided scientific guidance. QS and CW wrote the manuscript. All authors reviewed the manuscript.

## Conflict of Interest

The authors declare that the research was conducted in the absence of any commercial or financial relationships that could be construed as a potential conflict of interest.
